# Risk factors related to age at diagnosis of pancreatic cancer: a retrospective cohort pilot study

**DOI:** 10.1186/s12876-022-02325-7

**Published:** 2022-05-14

**Authors:** Ro-Ting Lin, Pei-Lung Chen, Chi-Ying Yang, Chun-Chieh Yeh, Chun-Che Lin, Wen-Hsin Huang, An-Ko Chung, Jaw-Town Lin

**Affiliations:** 1 Department of Occupational Safety and Health, College of Public Health, China Medical University, Taichung, 406040 Taiwan; 2 Department of Medical Genetics, National Taiwan University Hospital, Taipei, 100226 Taiwan; 3 Graduate Institute of Medical Genomics and Proteomics, National Taiwan University, Taipei, 100025 Taiwan; 4 Department of Internal Medicine, Digestive Medicine Center, China Medical University Hospital, Taichung, 404332 Taiwan; 5 Department of Surgery, China Medical University Hospital, Taichung, 404332 Taiwan; 6 Department of Surgery, Asia University Hospital, Taichung, 413505 Taiwan; 7 Division of Gastroenterology and Hepatology, Department of Internal Medicine, E-Da Hospital, No. 1, Yida Rd., Yanchao Dist., Kaohsiung, 824005 Taiwan

**Keywords:** Age at diagnosis, Pancreatic cancer, Risk factors, Alcohol drinking, Genetics

## Abstract

**Background:**

Increased pancreatic cancer incidence has been observed among younger than in older adults. This pilot study aimed to determine the feasibility of a large study that would compare the age at diagnosis of pancreatic cancer among patients with different risk factors.

**Methods:**

We compared the age at diagnosis of pancreatic cancer between groups of pancreatic cancer patients exposed and not exposed to the identified risk factors. We estimated the age at which exposure started, average exposure quantity, and total years of exposure and investigated their relationships with age at diagnosis of pancreatic cancer.

**Results:**

Sixteen out of 24 (67%) subjects carried known genetic factors and/or had smoking and/or drinking habits; however, an earlier age of pancreatic cancer diagnosis was not observed. Conversely, we found a significant correlation between the age at which alcohol consumption was started and the age at diagnosis of pancreatic cancer (*r* = 0.8124, *P* = 0.0043).

**Conclusions:**

Our pilot study suggested that a large study following this study design is feasible and that the following should be conducted in a large study: mediation analysis for disease-related factors, advanced genomic analysis for new candidate genes, and the correlation between age of first exposure to risk factors and pancreatic cancer onset.

**Supplementary Information:**

The online version contains supplementary material available at 10.1186/s12876-022-02325-7.

## Background

Pancreatic cancer has become the fifth leading cause of cancer-related deaths worldwide [1], causing more than 531,000 deaths in 2019 [2]. Between 2000 and 2019, the global trends in pancreatic cancer death rates increased by 42% [1]. Two-thirds of pancreatic cancer mortalities occurred in those aged 65 years and older [1]. Nevertheless, a greater increase in pancreatic cancer incidence was observed among younger adults than in the other age groups, especially those aged 15–34 years [3]. Thus, identifying factors related to the age at pancreatic cancer onset can contribute to effectively allocating prevention resources.

The risk factors for pancreatic cancer are complex and multifactorial [4–6]; that is, different age groups have different biological and socioeconomic effects. Past research has shown that genetic factors, smoking, and alcohol consumption may lower the age of pancreatic cancer development [7–11]. In familial pancreatic cancer kindreds and smokers, pancreatic cancer developed approximately 3.7–10 years earlier than that in non-smokers [12, 13]. Different patterns of *CDKN2A* mutation and increased gene expression of *FOXC2* was found in early-onset pancreatic cancer cases [14]. A multicenter study further showed the age of diagnosis varied with the youngest age in those who smoked greater than one pack of cigarette per day (63.8 years), older in those who smoked less than one pack of cigarette per day (66.5 years), to oldest in non-smokers (69.5 years) [15]. Similar but smaller differences were found in Japan, with an estimated onset of 3.5 years earlier in those smoking 20 cigarettes or more per day (65.9 years) than in non-smokers (68.6 years) [16]. The Japanese study also found an earlier onset of pancreatic cancer among alcohol drinkers who consumed > 80 g per day (63.8 years) than those who consumed < 50 g per day (68.0 years) [16].

These findings highlight the importance of the status (e.g., with or without genetic factors, smoking or not, drinking or not) and magnitude (e.g., number of cigarettes smoked per day or volume of alcoholic drink per day) of exposure on the age at diagnosis of pancreatic cancer [17, 18]. Beyond this, recent research has shown that starting smoking at a younger age was associated with increased rate ratios of lung cancer and all cancer mortality compared with that in non-smokers [19–21]. This evidence supports the hypothesis that the initial time of exposure to risk factors is also associated with the age of pancreatic cancer onset. However, whether the age of regular smoking and drinking onset is associated with younger age at diagnosis of pancreatic cancer remains unclear. In addition, the duration of exposure (e.g., number of years of smoking and drinking) to these factors should be considered in studies.

Establishing a pancreatic cancer cohort with a diverse set of risk factors and life course exposure profiles can help elucidate the correlation between exposure and the age of pancreatic cancer incidence. To assess the feasibility of a large study [22], we conducted this pilot study to determine the proportion of patients with pancreatic cancer exposed to the risk factors of interest. In this pilot study, we also aimed to compare the age at diagnosis of pancreatic cancer among patients with different genetic, lifestyle, and disease-related factors.

## Methods

This was a retrospective cohort pilot study in which we recruited hospitalized patients with suspected pancreatic cancer at a medical center, the China Medical University Hospital, in Taiwan. Potential subjects were those admitted to digestive health clinics, hematology and oncology clinics, and surgical clinics of China Medical University Hospital between July 1, 2020, and December 10, 2020. Given that the risk factors for pancreatic cancer in young adults were one of our main research interests, we included all potential subjects aged < 60 years in this study. For those aged 60–69 years and > 70 years, we sampled 75% and 50% of potential subjects for further assessment. The inclusion criteria were subjects with a final diagnosis of pancreatic cancer (C25.0–C25.9, based on the International Statistical Classification of Diseases and Related Health Problems, 10th revision [ICD-10]). The exclusion criteria were subjects without complete biospecimens and questionnaire data. Only subjects who signed the informed consent form were included in the data collection and interviews. The study was approved by the Research Ethics Committee III of the China Medical University and Hospital (CMUH109-REC3-026).

### Blood sample collection and germline DNA genotyping

For the eligible participants, blood samples (8–10 c.c.) were obtained using lavender top tubes containing ethylenediaminetetraacetic acid (EDTA), centrifuged to separate the buffy coat, and then stored in a –80 °C refrigerator. After collecting all the target samples, they were sent to a genotyping laboratory for DNA extraction. When all target samples were collected, we sent them to a genotyping laboratory for DNA extraction. Next, whole-genome sequencing (WGS) was performed with genomic DNA on the Illumina NovaSeq with 2 × 150 bp paired-end reads. The sequencing analysis pipeline was conduct using GATK Best Practices workflow (v4.1.8.0) [23]. Sequencing reads were mapped to human reference genome GRCh38 using Burrows–Wheeler aligner (BWA) software [24], and the aligned bam files were processed with markduplicates and base recalibration. The germline variants were called using HaploTypeCaller in genomic variant call format (GVCF) mode, and all GVCF were merged using GenomicsDBImport, followed by joint genotyping using GenotypeGVCFs. The variants were annotated using ANNOVAR [25] and were interpreted into five classes (pathogenic, likely pathogenic, uncertain significance, likely benign, and benign) according to the American College of Medical Genetics and Genomics (ACMG) guidelines [26] through the TAIGenomics platform (https://www.taigenomics.com) and manually curation. The variants with high allele frequencies in Genome Aggregation Database (gnomAD, https://gnomad.broadinstitute.org) database or Taiwan biobank (https://taiwanview.twbiobank.org.tw/index) database were filtered out. The pathogenic or likely pathogenic variants in pancreatic cancer predisposition genes including DNA repair, pancreatitis, cell growth and cell mobility (Additional file 1: Table S1) were further selected and were checked using Integrative Genomics Viewer [27].

### Epidemiological data

Epidemiological data were retrospectively collected via face-to-face interviews. The self-reported questionnaire collected data on age (defined as age at the interview date), sex, past medical history, family health history, dietary habits, lifestyle, and exposure to chemicals. Past medical history was composed of four items, including four questions on the diagnosis of cholelithiasis, pancreatitis, diabetes, and any cancer history (for example, “Have you ever been diagnosed with pancreatitis? Yes/No.”). If patients reported “Yes,” we further inquired about the year and month of diagnosis. Family health history was composed of two items about any family member diagnosed with diabetes and/or cancer (for example, “Do you have any family member with a cancer diagnosis? Yes/No.”). If patients reported “Yes,” we further inquired about their relationship with the patients and the type of cancer. Dietary habits recorded the frequency and volume of consuming high-fat and/or oily foods (e.g., pork belly, pork rinds, popcorn chicken, French fries, etc.) and sugary foods (e.g., dessert and drinks). The recall period of dietary habits corresponds to the previous one month and two years, respectively, and the response scale ranged from not at all (i.e., vegetarian), fewer than one portion (a fist size) per month, to one or more portions per day. Lifestyle factors included cigarette smoking and alcohol consumption. The recall period of lifestyle factors corresponded to three periods: aged < 20 years, aged 20–39 years, and aged ≥ 40 years. We recorded the age of regular smoking and drinking duration, average weekly consumption volume (excluding tastes and sips) in each period, and age at quitting smoking and drinking (if applicable). Chemical exposure recorded the types of chemical substances (e.g., pesticides) and frequency of exposure (e.g., times or hours per week). The recall period of chemical exposure corresponded to their job and living history since birth.

### Data analysis

We calculated the distribution of risk factors among the patients. Continuous variables were expressed as mean with standard deviation (SD) as well as median with interquartile range (IQR). For the categorical variables, we expressed the number of subjects as percentages. For each risk factor, subjects were divided into two groups: one with exposure and the other without exposure. We used the Mann–Whitney U test to compare the age at diagnosis of pancreatic cancer between the groups. The risk factors were divided into three or more groups, and we used the Kruskal–Wallis test to compare age at diagnosis of pancreatic cancer among the groups. For smoking and drinking, we calculated the age at which exposure started, average consumption volume per week, and total years of exposure, and we used the Spearman rank-order correlation coefficient to measure their relationships with age at diagnosis of pancreatic cancer. All statistical analyses were performed using SAS version 9.4 (SAS Institute, Cary, NC, USA).

## Results

Between July 1, 2020, and December 10, 2020, 43 patients with suspected pancreatic cancer were hospitalized (Additional file 2: Figure S1). After being informed of the study purpose, these 43 subjects were assigned to different age groups (18 in the group aged < 60 years, 12 in the group aged 60–69 years, and 13 in the group aged ≥ 70 years). A total of 34 subjects were sampled from each group (18 aged < 60 years, nine aged 60–69 years, and seven aged ≥ 70 years). Before the eligibility assessment, the investigators did not exclude any potential subjects because some hospitalized patients might need an emergency operation for pain relief. After the eligibility assessment, a total of 27 subjects fulfilled the inclusion criteria, among which 24 had complete data on biospecimen and a questionnaire for analysis.

Table 1 summarizes the characteristics of all the subjects derived from the questionnaire survey and WGS. The mean age of participants was 58.7 years (SD = 13.3; range, 36.1–82.5 years), and 19 (79.2%) were males, and five (20.8%) were females. Most participants (n = 13 [54.1%]) had lesions located in the head of the pancreas (C25.0), followed by the tail (C25.2: n = 7 [29.2%]) and the body (C25.1: n = 3 [12.5%]); only one participant had lesions located in both the head and body of the pancreas and was coded as C25.9. Six participants (25.0%) had either cholelithiasis, pancreatitis, or diabetes for three or more years before the diagnosis of pancreatic cancer. Most participants (19 [79.2%]) had at least one first-degree relative with diabetes, cancer, or both. Among them, two had family members diagnosed with pancreatic cancer. The proportions of subjects with smoking habits, drinking habits, and chemical exposures were 58.3% (n = 14), 41.7% (n = 10), and 37.5% (n = 9), respectively. Ten participants (41.7%) frequently consumed high-fat and/or oily foods, sugary desserts and/or drinks, or both.

**Table 1:** Demographics of all the participants

Variables	Participants (n = 24)
*Age*	
Mean (SD), years	58.7 (13.3)
Median (IQR), years	56.7 (20.5)
*Sex*	
Male	19 (79.2%)
Female	5 (20.8%)
*Final diagnosis, ICD-10 codes*	
Malignant neoplasm of head of pancreas, C25.0	13 (54.1%)
Malignant neoplasm of body of pancreas, C25.1	3 (12.5%)
Malignant neoplasm of tail of pancreas, C25.2	7 (29.2%)
Malignant neoplasm of pancreas, unspecified, C25.9	1 (4.2%)
*Past medical history*	
Cholelithiasis*	2 (8.3%)
Pancreatitis*	1 (4.2%)
Diabetes*	3 (12.5%)
Cancer, except for pancreatic cancer	3 (12.5%)
*Family health history*^†^	
Diabetes	12 (50.0%)
Cancer, all types	8 (33.3%)
Cancer, pancreas	2 (8.3%)
*Deleterious germline mutations*	
Yes	5 (20.8%)
*Cigarette smoking*	
Past	6 (25.0%)
Current	8 (33.3%)
*Alcohol consumption*	
Past	4 (16.7%)
Current	6 (25.0%)
*Chemical exposure*	
Yes	9 (37.5%)
*Dietary habit*	
Frequent^‡^ consumption of high-fat or oily foods	5 (20.8%)
Frequent^‡^ consumption of sugary desserts and drinks	8 (33.3%)

*ICD-10* International Statistical Classification of Diseases and Related Health Problems, 10th revision; *IQR* interquartile range; *SD* standard deviation (SD)

*Participants with cholelithiasis, pancreatitis, and/or diabetes for three or more years before the diagnosis of pancreatic cancer

^†^Any one of first-degree relatives, including the study participant’s parents, siblings, and children

^‡^Frequent is defined consuming at three or more portions per week

Given that genetic variants are risk factors for pancreatic cancer, we performed whole genome sequencing for 24 pancreatic cancer patients. After read deduplication, the sequence depth was approximately 30 × per sample. The identified variants were interpreted and classified into five-classes (pathogenic, likely pathogenic, uncertain significance, likely benign, and benign) according to the ACMG guidelines [26]. Herein we report variants classified as pathogenic or likely pathogenic in a virtual panel of 34 genes (Additional file 1: Table S1) related to DNA repair, pancreatitis, cell growth and cell mobility [28–30]. Overall, the five heterozygous pathogenic variants were identified in five patients (5/24; 20.8%), and those variants were predicted as loss of function including 2 frameshift indels, 2 splicing variants and 1 stopgain variant. Four of them are in DNA repair genes including *BRCA1*, *BRCA2*, *ATM* and *POLQ*; one of which, a *BRCA2* variant (NM_000059.4:c.7977-1G > T), was previously reported as pathogenic variant in ClinVar database (Allele ID: 131701) (https://www.ncbi.nlm.nih.gov/clinvar). The 5th variant is in a pancreatitis gene *SPINK1*.

Table 2 lists each subject’s characteristics regarding whether they carried a certain risk factor category or not. We observed that 23 (95.8%) subjects had at least one of the listed risk factors, broadly categorized as genetic, disease-related, and lifestyle factors, except for the youngest subject (PC015). Most subjects had either one (n = 12 [50.0%]) or two (n = 10 [41.7%]) risk categories. There was only one subject that had none of the identified risk categories (n = 1 [4.2%]), and another subject had three risk categories (n = 1 [4.2%]). Specifically, six out of the 24 subjects (25%) were found to be related to genetic factors, i.e., either familial pancreatic cancer (n = 1), germline mutation (n = 4), or both (n = 1). Totally, 67% of subjects (n = 16) carried known genetic factors and/or had smoking and/or drinking habits. Subjects with cholelithiasis, pancreatitis, or diabetes were also exposed to other lifestyle factors before the onset of these diseases. For example, all pancreatitis and diabetes patients smoked regularly 20 years or more before the diagnosis of diabetes.

**Table 2:** Characteristics of individual participants

Subject ID	Sex	Age at diagnosis with pancreatic cancer	Genetic factors		Disease–related factors				Lifestyle factors				
			Variants on recognized genes	Family history of cancer in FDR	Cholelithiasis (age of diagnosis)	Pancreatitis (age of diagnosis)	Diabetes (age of diagnosis)	Other types of cancer	Cigarette smoking	Alcohol consumption	Chemical exposure	Frequent^†^ consumption of high-fat or oily foods	Frequent^†^ consumption of sugary foods
PC002	M	74.3	–	–	No	No	No	–	Past	No	No	No	No
PC003	M	82.5	–	–	No	No	Yes (58.5)	Colon cancer	Past	No	No	No	Yes
PC004	F	54.3	–	–	No	No	Yes (53.0)	No	Current	Current	No	No	No
PC005	M	41.1	–	No	Yes (40.2)	No	No	No	No	No	No	Yes	No
PC006	M	43.8	*BRCA2*	No	No	Yes (43.5)	No	No	Current	Current	Yes	No	No
PC008	M	66.3	–	Yes*	No	No	No	No	No	No	No	No	No
PC009	F	47.4	*SPINK1*	No	No	Yes (47.1)	No	Thyroid cancer	Current	Current	Yes	Yes	Yes
PC010	M	67.2	–	Yes	No	No	No	No	No	No	Yes	No	No
PC011	M	77.4	–	No	Yes (62.4)	No	No	No	No	No	Yes	No	No
PC012	M	55.5	–	Yes	No	No	Yes (48.3)	No	Past	No	No	Yes	Yes
PC013	M	64.3	–	No	No	No	No	No	No	No	Yes	No	Yes
PC014	M	67.2	*–*	No	No	Yes (39.6)	No	No	Past	Past	No	Yes	No
PC015	M	36.1	–	No	No	No	No	No	No	No	No	No	No
PC017	M	47.5	–	No	No	No	No	No	Past	Past	No	Yes	Yes
PC018	M	74.2	*ATM*	Yes	No	No	No	No	No	Past	Yes	No	No
PC019	F	77.7	–	Yes	Yes (67.7)	No	Yes (77.3)	No	No	No	Yes	No	No
PC023	M	54.9	–	Yes	No	No	No	No	Current	Current	No	No	Yes
PC031	M	49.9	–	No	No	No	Yes (42.6)	No	Current	No	No	No	No
PC032	M	56.2	–	No	No	No	No	No	Current	Current	No	No	No
PC034	F	57.1	–	Yes	No	No	No	Breast cancer	No	No	No	No	No
PC035	M	68.5	*POLQ*	No	No	No	No	No	Past	No	Yes	No	No
PC037	M	60.1	*BRCA1*	Yes*	No	No	No	No	Current	Current	No	No	No
PC038	F	44.3	–	No	No	No	No	No	No	No	No	No	Yes
PC043	M	40.8	–	No	No	No	No	No	Current	Past	Yes	No	Yes

*FDR* first-degree relative subject's parents (father or mother), siblings (brother or sister), and children

^*^FDR was diagnosed with pancreatic cancer

^†^Frequent is defined consuming at three or more portions per week

**Fig. 1:**
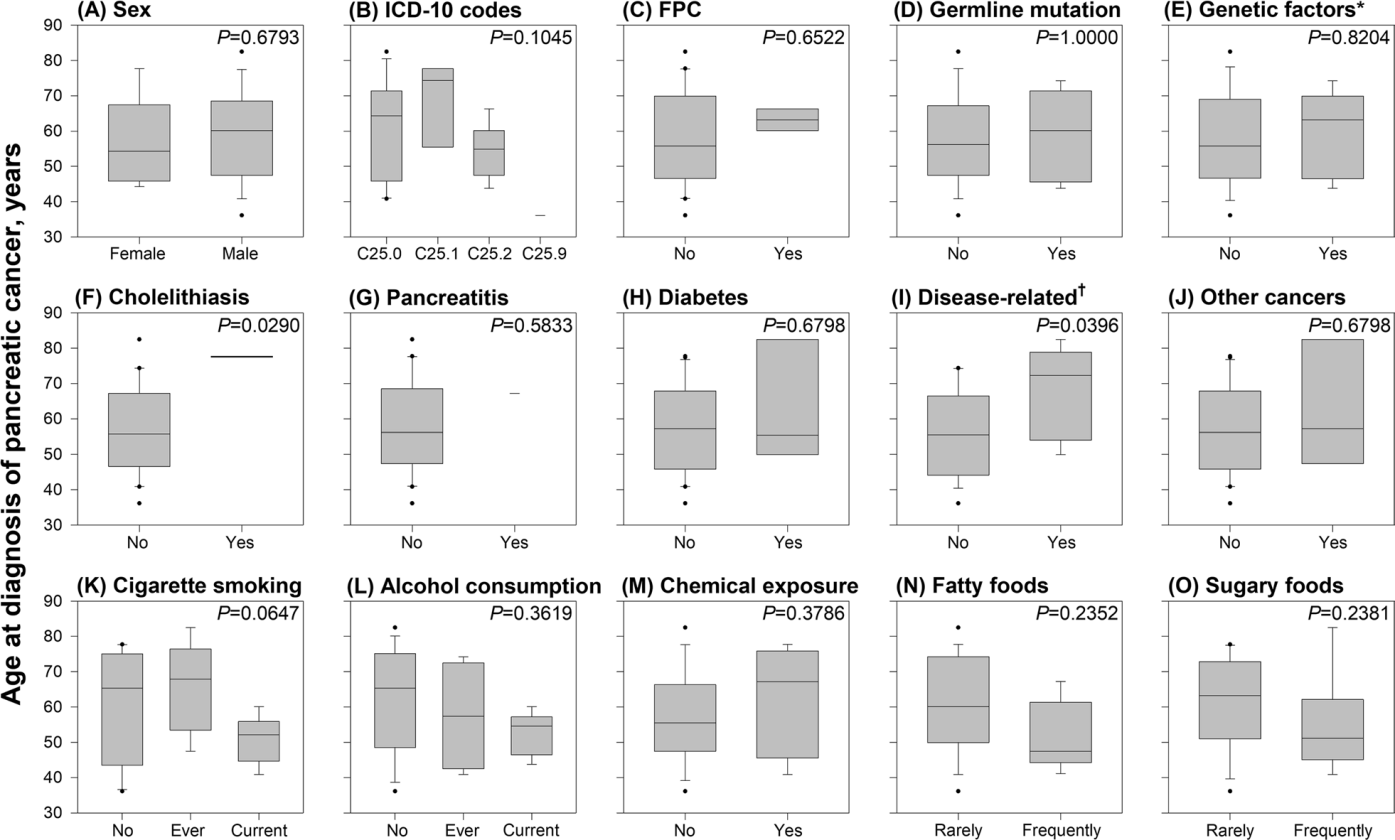
Mean age at diagnosis of pancreatic cancer by sex, codes, genetic, disease-related, and lifestyle factors. *FPC* Familial pancreatic cancer, defined as participants with first-degree relative diagnosed with pancreatic cancer; *ICD-10* International Statistical Classification of Diseases and Related Health Problems, 10th revision. *Participants with either familial pancreatic cancer, germline mutation, or both. †Participants with cholelithiasis, pancreatitis, and/or diabetes for three or more years before the diagnosis of pancreatic cancer

**Fig. 2:**
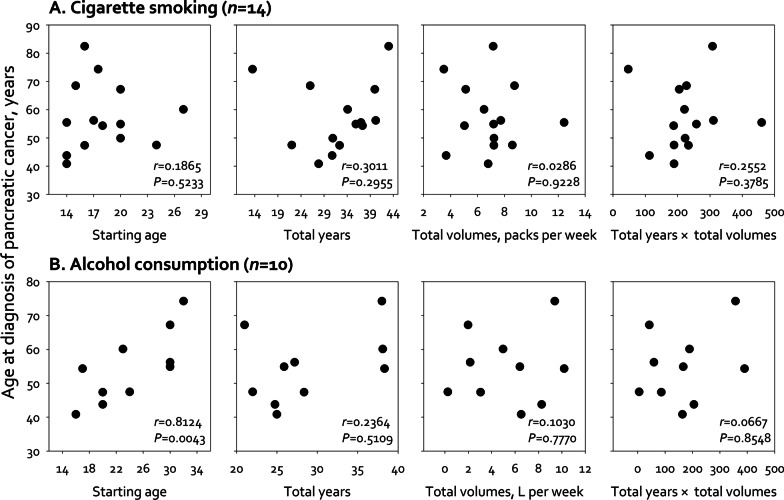
Exposure age, consumption, and duration, and their relationships with age at diagnosis of pancreatic cancer. *L* liter; *n* number of participants; *P p*-value; *r* correlation coefficient

We tested the age at diagnosis of pancreatic cancer between those with and without exposure to a specific risk factor. For the categorical risk factors, we did not find significant differences in age at diagnosis between groups (Fig. 1), except for patients with cholelithiasis who had a later age of pancreatic cancer onset than those without cholelithiasis (*P* = 0.0290). However, for the continuous risk factors (i.e., age, average consumption volume per week, and total years of exposure to smoking and drinking) (Fig. 2), we found a significant correlation between the age at which alcohol consumption was started and the age at diagnosis of pancreatic cancer (*r* = 0.8124, *P* = 0.0043).

## Discussion

Younger patients with pancreatic cancer are healthier and tend to receive aggressive treatments related to better survival than older patients [31, 32]. However, young adults without health problems are usually less likely to seek medical care or undergo health examinations as frequently as elders. Therefore, young patients with pancreatic cancer are more likely to be diagnosed at an advanced stage, as observed in a population-based study in the United States [33]. Understanding the effect of risk factors on different age groups can be critical in developing an effective intervention.

Our pilot study incorporated an in-depth survey of historical exposure to risk factors and their exposure time for 24 patients with pancreatic cancer to assess the feasibility of a large study. We used the global averages to determine the proportion of patients with pancreatic cancer who were exposed to risk factors of interest, which could increase the generalizability. Our results show that two-thirds of the subjects carried known genetic factors and/or had smoking and/or drinking habits. All proportions of exposure to genetic factors, cigarettes, and alcohol reached the expected values. As for the correlation with age at diagnosis of pancreatic cancer, we did not observe an earlier age of pancreatic cancer incidence among pancreatic cancer patients who carried germline mutations or had a family history of pancreatic cancer. Conversely, we observed that subjects who started drinking at a younger age had an earlier pancreatic cancer incidence than those who started drinking at an older age. These findings suggest that a large study, following the study design of this pilot study, is feasible and worthy to be conducted in the future.

Disease-related factors, including cholelithiasis, pancreatitis, and diabetes, have been regarded as risk factors and consequences of pancreatic cancer [34, 35]. However, short time windows between diagnosis of these disease-related factors and diagnosis of pancreatic cancer prevent these factors from becoming suitable early predictors of pancreatic cancer. For example, individuals first meeting the glycemic criteria for new-onset diabetes after age 50 had a six- to eight-fold higher risk of being diagnosed with pancreatic cancer, compared with the general population, but it was within only three years [36]. In addition, approximately 15% of participants diagnosed with chronic pancreatitis had developed diabetes within the previous 36 months [37]. The time between the diagnosis of these disease-related factors and pancreatic cancer was too short. Therefore, these disease-related factors are more like early signs of pancreatic cancer rather than carcinogenic factors. To differentiate the time of events, we defined exposure to these factors three or more years before the diagnosis of pancreatic cancer. With such a definition, we were able to observe that disease-related factors, including cholelithiasis, pancreatitis, and diabetes, did not exist alone, and they usually appeared 20 years or more after exposure to lifestyle factors. Moreover, we observed that patients with cholelithiasis had a later age of pancreatic cancer onset than those without cholelithiasis. This pilot study also suggests that these disease-related factors can be considered as mediators in the tumorigenesis process in a large study. Analyzing data with sufficient time intervals and information regarding time intervals among moderating factors (e.g., lifestyle and environmental factors), mediating factors (e.g., disease-related factors), and pancreatic cancer may fill the knowledge gap regarding the critical timing of pancreatic tumorigenesis.

Recent genomic studies suggest that the early onset of pancreatic cancer may be related to gene mutations [38, 39]. Individuals with genetic risk factors may also be more susceptible to lifestyle and environmental factors, which may further promote pancreatic tumorigenesis [7, 12, 40]. We did not observe a significant difference in age at diagnosis of pancreatic cancer between patients with and without known genetic factors. Instead, we found a significant correlation between early age at alcohol consumption and early age at diagnosis of pancreatic cancer. Our findings were consistent with Anderson and colleagues’ findings that only a non-statistically significant trend was observed for family history of pancreatic cancer after adjusting for covariates, such as the amount of alcohol use [15]. As suggested by Klein, pancreatic cancer patients from the same families can be attributed to both genetic predispositions and shared environmental factors [34]. We expected that with the advancement in genomic technology in our large study, more candidate genes associated with pancreatic cancer could be identified. Moreover, novel genetic analysis may contribute to the identification of new factors that can help explain the cause of the youngest patients (PC015) in our pilot study.

Although only 10 subjects were past or current alcohol drinkers in our pilot study, a significant correlation between age at alcohol consumption and age at diagnosis of pancreatic cancer was observed. Early age at drinking has been reported to increase the risk of alcohol intoxication and alcohol dependency, especially in people who started to drink before age 20 years [41]. Hingson et al. found that these young people were more likely to develop alcohol dependence within 10 years of drinking onset, and two-thirds of them experienced it before the age of 25 years [41]. The ten subjects with drinking experience in our pilot study had a mean age of the onset of drinking at 24.2 years (range, 16.0–32.0 years) and a mean duration of 28.9 years (range, 21.0–38.3 years). The average consumption volume of alcohol was 5319 c.c. per week (range, 245–10,186 c.c. per week). Among these ten subjects with drinking experience, four started drinking before or at the age of 20 years (average, 18.3 years; range, 16.0–20.0 years), and six started after 20 years old (average, 28.2 years; range, 23.0–32.0 years). The average consumption volume of alcohol in the four subjects drinking at ≤ 20 years old was 7006 c.c. per week (range, 3047–10,186 c.c. per week), approximately 1.7-fold of the volume in the six subjects drinking after 20 years old (average, 4195 c.c. per week; range, 245–9395 c.c. per week). In terms of quitting alcohol, there was only one out of four subjects started drinking at ≤ 20 years old (25%), whereas three out of six subjects started drinking at > 20 years old (50%). Etiologically, the underlying mechanism by which alcohol promotes pancreatic carcinogenesis may exert by alcohol metabolites and through the inflammation pathway [42]. Early exposure to alcohol at a young age represents the exposure of pancreas to toxic alcohol metabolites during its more susceptible period. Based on our preliminary data, those with earlier age at drinking onset were also heavier alcohol consumers and had a low proportion of alcohol quitting. Excessive and persistent drinking of alcohol represents nonstop damage to the pancreas. Our pilot study did not aim to test a hypothesis using a sample size of ten pancreatic cancer patients nor make inferences on non-pancreatic cancer patients. Nonetheless, under such a small sample size, we detected a potential correlation between age at exposure and age at diagnosis of pancreatic cancer. Our pilot study suggests that a large study should elucidate the interaction between the magnitude of exposure (e.g., the volume of alcohol drinking), duration of exposure, and age of first exposure. In order to validate the potential dose–response correlation between the age at which alcohol consumption was started and the age at diagnosis of pancreatic cancer and risk factors, the large study is planned with six dose groups based on age at alcohol consumption (3 years increment for each dose). A sample size of 12 should be sufficient using a two-tailed test with 95% power and a 5% level of significance [43]. When further considering sex (two groups), genetic (two groups), disease-related (two groups), and other lifestyle factors (two groups), the proposed large study may require recruiting 270 potential pancreatic cancer subjects and including 192 confirmed cases in the data analysis. Factors that may reduce the number of recruited subjects should be avoided in the protocol. Consequently, we proposed the refined protocol to recruit all potential samples instead of applying different sampling rates for different age groups (i.e., removing the sampling procedure from Step 3 in Additional file 2: Figure S1).

Our pilot study demonstrated the feasibility of a large study based on this study design. Some limitations should be noted when interpreting the results of this pilot study. First, the small sample size limits the ability to estimate the effect size, despite the fact that the sample size is not usually required for pilot studies [22]. The significant finding under such a small sample size warrants further analysis in a large study. Second, although our patients responded to the starting age and amount of exposure to risk factors, recall bias may exist. Earlier experiences are more likely to be biased than they are in their later lives. Respondents may recall better if their exposure could be linked to experience in specific events such as smoking in the first year of high school. To reduce recall bias, information on the exposure profile was collected via an in-depth interview by trained nurses to assist the patients in connecting to a specific life event that they could remember the years qualitatively. Qualitative data obtained from interviews can be analyzed in a large study.

The main strength of our pilot study is to present the design of a retrospective cohort that collected data on pancreatic cancer patients exposed to a diverse set of risk categories encompassing genetic, disease-related, and lifestyle factors. Studies without such comprehensive and longitudinal data may not be able to elucidate the critical exposure or provide implausible estimates of relative risks, although they may have aggregated data on a large sample size. In contrast, through in-depth interviews with each patient about their lifestyles and disease history, given that the patients could remember, we were able to obtain estimates of time windows of exposure, specifically, the age started exposure, duration, and intensity of cigarette smoking and alcohol consumption.

## Conclusions

In conclusion, our pilot study incorporated an in-depth survey of historical exposure to risk factors and their exposure time for 24 patients with pancreatic cancer and suggested that a large study following the study design of this pilot study is feasible. We determined that our large study would be feasible to carry on if the following criteria were fulfilled: (1) the proportion of eligible patients who carried genetic factors was 10% or more [44]; (2) the proportion of eligible patients who regularly smoked cigarettes was 32.7% and more [45]; (3) the proportion of eligible patients who regularly drank alcohol was 32.5% or more [46]; and (4) the correlations between exposure to risk factors and age at diagnosis of pancreatic cancer were observed. This pilot study also suggests that the following three analyses should be conducted in a large study: mediation analysis for disease-related factors, advanced genomic analysis for new candidate genes, and the correlation between age of first exposure to risk factors and pancreatic cancer onset. The understanding of early life exposure may contribute to identifying useful indicators for the early detection of pancreatic cancer.

## Supplementary Information

**Additional file 1: Table S1**. Pancreatic cancer panel.

**Additional file 2: Figure S1**. Flow chart of recruitment of study subjects, sampling, eligibility assessment, data collection, and data analysis.

## Data Availability

The datasets generated and/or analysed during the current study are provided in Table 2. Other non-public datasets, including genomic data, are not publicly available due to the non-public data should not be shared publicly as participants were informed at the time of providing consent that only researchers involved in the project would have access to the original information they provided for the protection of personal privacy and patient’s right as well as compliance with research ethics. Non-public data are available from the corresponding author under the approvals of patients and the Research Ethics Committee III of the China Medical University and Hospital.

## Abbreviations

GVCF: Genomic variant call format
ICD-10: International Statistical Classification of Diseases and Related Health Problems, 10th revision
IQR: Interquartile range
SD: Standard deviation
WGS: Whole-genome sequencing
