# Supplementary material for: Risk factors related to age at diagnosis of pancreatic cancer: a retrospective cohort pilot study
Source: BMC Gastroenterol. 2022 May 14;22:243. doi: 10.1186/s12876-022-02325-7 (PMC9107247; doi:10.1186/s12876-022-02325-7)
Supplement: Supplementary file 1 — Additional file 1: Table S1. Pancreatic cancer panel. [file 12876_2022_2325_MOESM1_ESM.pdf]

Supplementary Table S1. Pancreatic cancer panel

GENE	PATHWAY	GENOMIC REGION (GRCH38)	REFSEQ (MANE SELECT)
CFTR	Pancreatitis susceptibility	chr7:117287120-117715971	NM_000492.4
PRSS1	Pancreatitis susceptibility	chr7:142749468-142753076	NM_002769.5
SPINK1	Pancreatitis susceptibility	chr5:147824572-147831671	NM_001379610.1
GGT1	Pancreatitis susceptibility	chr22:24594811-24629005	NM_001288833.2
CTRC	Pancreatitis susceptibility	chr1:15438442-15449242	NM_007272.3
CPA1	Pancreatitis susceptibility	chr7:130380339-130388114	NM_001868.4
CPB1	Pancreatitis susceptibility	chr3:148791102-148860187	NM_001871.3
BARD1	DNA repair	chr2:214725646-214809683	NM_000465.4
BRCA1	DNA repair	chr17:43044295-43170245	NM_007294.4
BRCA2	DNA repair	chr13:32315086-32400268	NM_000059.4
FANCA	DNA repair	chr16:89737549-89816657	NM_000135.4
FANCC	DNA repair	chr9:95099054-95426796	NM_000136.3
FANCM	DNA repair	chr14:45135930-45200890	NM_020937.4
MLH1	DNA repair	chr3:36993350-37050846	NM_000249.4
MSH2	DNA repair	chr2:47403067-47663146	NM_000251.3
MSH6	DNA repair	chr2:47695530-47810063	NM_000179.3
NBN	DNA repair	chr8:89933331-90003228	NM_002485.5
PALB2	DNA repair	chr16:23603160-23641310	NM_024675.4
FANCG	DNA repair	chr9:35073835-35079942	NM_004629.2
EPCAM	DNA repair	chr2:47345158-47387601	NM_002354.3
POLN	DNA repair	chr4:2071918-2242121	NM_181808.4
POLQ	DNA repair	chr3:121431431-121545988	NM_199420.4
MUTYH	DNA repair	chr1:45329163-45340893	NM_001048174.2
PMS2	DNA repair	chr7:5970925-6009106	NM_000535.7
ATM	DNA repair	chr11:108223044-108369102	NM_000051.4
CDKN2A	Cell growth	chr9:21967752-21995301	NM_000077.5
KRAS	Cell growth	chr12:25205246-25250936	NM_004985.5
SMAD4	Cell growth	chr18:51028394-51085045	NM_005359.6
PTEN	Cell growth	chr10:87863625-87971930	NM_000314.8
TP53	Cell growth	chr17:7661779-7687538	NM_000546.6
STK11	Cell growth	chr19:1177558-1228431	NM_000455.5
CHEK2	Cell growth	chr22:28687743-28742422	NM_007194.4
APC	Cell mobility	chr5:112707498-112846239	NM_000038.6
PALLD	Cell mobility	chr4:168497052-168928457	NM_001166108.2
