# Supplementary material for: Risk factors related to age at diagnosis of pancreatic cancer: a retrospective cohort pilot study
Source: BMC Gastroenterol. 2022 May 14;22:243. doi: 10.1186/s12876-022-02325-7 (PMC9107247; doi:10.1186/s12876-022-02325-7)

Supplementary Figure S1. Flow chart of recruitment of study subjects, sampling, eligibility assessment, data collection, and data analysis

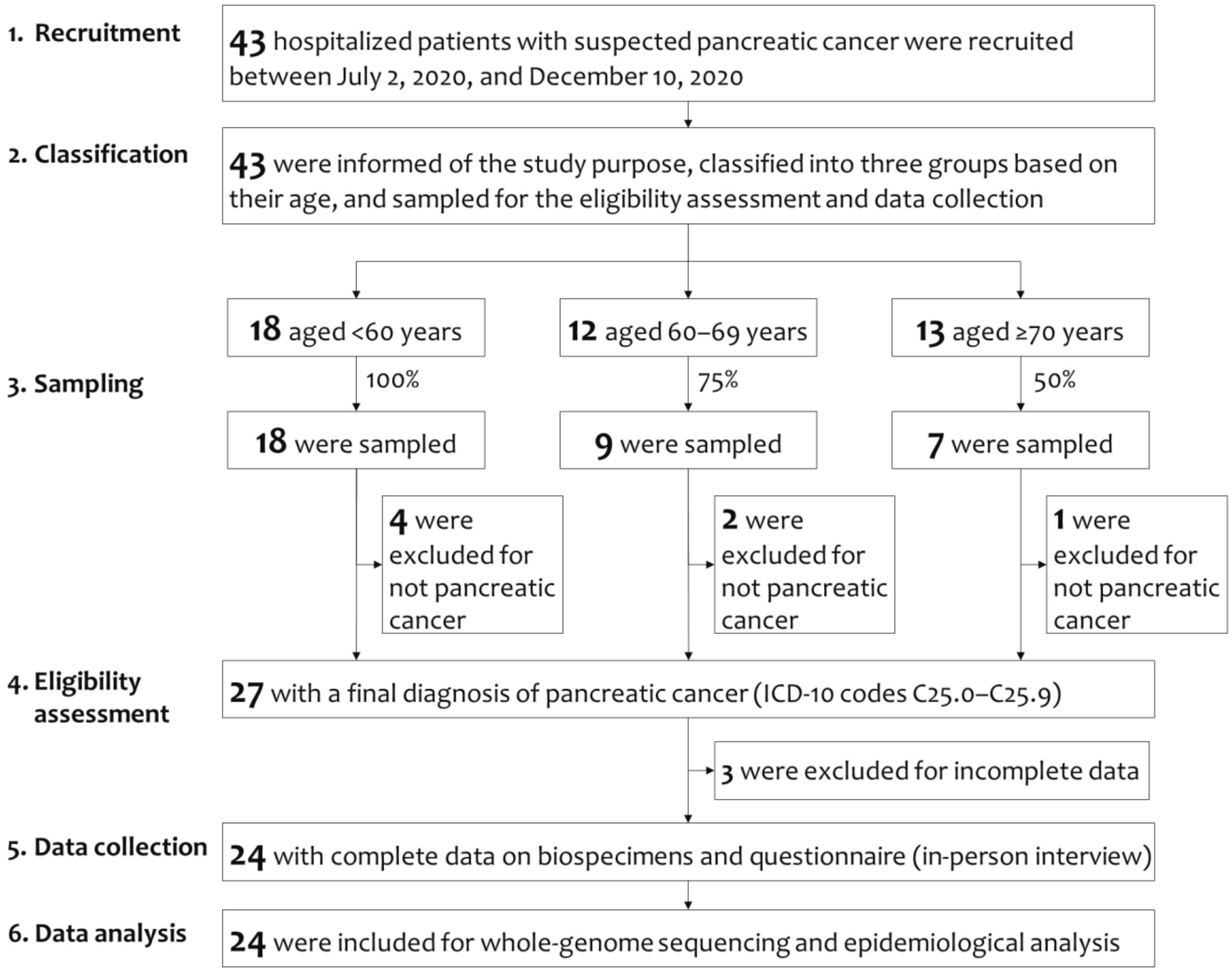

Supplement: Supplementary file 2 — Additional file 2: Figure S1. Flow chart of recruitment of study subjects, sampling, eligibility assessment, data collection, and data analysis. [file 12876_2022_2325_MOESM2_ESM.pdf]
